# Green Conversion of the Hazardous Cathode Ray Tube and Red Mud into Radiation Shielding Concrete

**DOI:** 10.3390/ma15155316

**Published:** 2022-08-02

**Authors:** M. I. Sayyed, Nouf Almousa, Mohamed Elsafi

**Affiliations:** 1 Department of Physics, Faculty of Science, Isra University, Amman 11622, Jordan; 2 Department of Physics, College of Science, Princess Nourah bint Abdulrahman University, P.O. Box 84428, Riyadh 11671, Saudi Arabia; nmalmousa@pnu.edu.sa; 3 Physics Department, Faculty of Science, Alexandria University, Alexandria 21511, Egypt; mohamedelsafi68@gmail.com

**Keywords:** hazardous wastes, cathode ray tube, gamma shielding factors, radiation shielding concrete

## Abstract

The present investigation was aimed at the utilization of alternate materials, emphasizing hazardous industrial products (red mud and cathode ray tubes), as constituents of radiation shielding concrete. The usage of these hazardous industrial products improves the sustainability and performance of the radiation shielding concrete. Five concrete blocks were cast and their density, compressive strength, gamma shielding factors, radiation absorption ratio, and transmission factor were explored. For this purpose, gamma-ray shielding measurements were done with the help of an HPGe detector. Mix-1, with zero contents of red mud and CRTs, had the lowest LAC. The LAC results demonstrated that the shielding performance of the current concretes would be better with the increase in red mud and cathode ray tube glass. The Transmission factor (TF) for the prepared concretes with a thickness of 2 cm varied between 11.9–26.1% at 0.06 MeV, while it varied between 4–13% for a thickness of 3 cm. The TF results showed that the composites with a thickness of 2, 3, or 5 cm are good shields against lower energy radiation. The radiation absorption ratio (RAR) for the prepared concretes is high at low energy, suggesting that these new composites can absorb most of the low-energy photons. The RAR results emphasize that the increase in CRTs in the new composites enhanced the radiation shielding features, and when the CRT glass is at a maximum, more attenuation was achieved.

## 1. Introduction

The use of gamma radiation has significantly advanced the fields of medicine, agriculture, mining, and other industries. However, exposure to gamma rays is of great concern, due to their high level of penetration and hazards, therefore, they must be minimized to the lowest possible level. Historically, gamma ray shielding materials employed lead as the element of choice for its high density and high atomic number. Worldwide, concrete comes in second place for gamma shielding efficiency, as it can be doped with lead and other high-Z elements. Alternatively, nontoxic, and lightweight lead-free materials are trending lately, where concrete composites can yield preferred properties unavailable in a single material [1,2,3,4,5].

With a basic premise of conserving virgin resources, the use of recycled materials and industrial by-products in shielding materials has been proposed. Modern engineered concrete can be processed to incorporate novel alternative materials to replace sand and aggregates of varying fine or coarse particles [6,7,8,9,10]. Examples of the secondary raw materials that were investigated are red clay, blast furnace slag, fayalite slag, fly ash, bottom ash, and waste glass [11,12]. Among these materials, waste glass has recently been proposed as a strong candidate to replace fine aggregates.

Great quantities of glass are contained in a variety of products, such as screens and lamps, so a couple of millions of tons of glass waste is sent to the landfill annually, worldwide. Additionally, glass possesses desirable properties, such as low permeability and superior hardness that can enhance the effectiveness of concrete as a shielding material [13]. Of particular interest when it comes to sustainability and recycling glass waste, is the cathode ray tube (CRT) glass which is being gradually replaced with advanced glasses such as LCDs and PDPs.

Cathode ray tubes are 85% glass and contain metals and oxides such as lead, cadmium, mercury, and other hazardous elements, which all are subject to recycling. Owing to the CRT’s chemical composition, specifically the presence of lead, recycling CRTs is optimized when employed in a gamma ray radiation shielding application, or nuclear waste encapsulation.

In addition to alternative aggregates, cement in concrete can be partially replaced with red mud, which is characterized by its high density and therefore has an enhanced potential to block gamma rays. Moreover, red clay is known for its high melting point and compressive strength. These two properties make red clay applicable for high-temperature resistance and high-strength shielding materials [14,15].

With a goal to preserve primary raw materials, the current study assesses the reuse of CRT glass in concrete as an alternative to fine aggregates. In combination with the CRT glass, red clay is proposed herein as an alternative to conventional sand in cement. The choice of CRT glass and red clay was determined by their effectiveness in shielding gamma rays. In this study, the various shielding properties of the resulting concrete are investigated experimentally and theoretically, encompassing linear and mass attenuation coefficients and mean free path. The study findings should provide baseline data on the shielding performance of this novel concrete composite.

## 2. Materials

### 2.1. Cement and Red Mud

We selected Portland cement (CEM II 42.5) in the current investigation since it has the following special features: a relative density of 3.15, compressive strength of 42.5 N/mm^2^, and surface area of 3556 cm^2^/g [16]. The red mud was collected from the city of Aswan in Egypt and crushed until it became almost the size of cement (using a sieve with a diameter of 50 µm) and dried. The density of the red mud is 3.26 g/cm^3^. The oxide compositions of both materials were tabulated in Table 1 using energy dispersive X-ray analysis (EDX analysis) model JSM-5300 JEOL type [17].

### 2.2. Aggregates

We used sand as a fine aggregate and limestone (with a diameter of 20 mm) as a coarse aggregate. Waste CRT glass (funnel and neck glass) was collected from a Toshiba color screen, ground to a fine powder, and then sieved through a 60-micro sieve. The funnel and neck of the CRT glass waste were selected due to their high lead content. The funnel is about 30% of CRT glass and has a lead content of about 22%, while the neck is about 5% of CRT glass and has a lead content of about 25%. In Table 2, we summarized the relative density and some physical properties of these aggregates. Moreover, using EDX analysis, we estimated the chemical composition of sand, stone, and CRT glass (see Table 3).

### 2.3. Chemical Additives

In this investigation, F-type super-plasticizer (SP) (relative density of 1.2 g/cm^3^) related to ASTM C494 [18] was utilized. This plasticizer was utilized to maintain slump stability in the fabricated samples.

### 2.4. Concrete Mix Design

The mix designs were developed according to the C&CI Design Method [19]. In Table 4, we list the proportions for a 1000 L mixture. The water to binder ratio is 0.5 for the current samples, while the proportions on the binder were 400 Kg/m^3^ with the replacement of cement with red mud at rates of 10%, 20%, 30%, and 40%. Similarly, the fine aggregate was replaced with CRT glass waste in the same proportions, respectively, with each mixture.

## 3. Methodology

### 3.1. Mechanical Test

According to BS1881: Part 116, we reported the compressive strength of cubed samples (with a length of 15 cm) after 28 days [20].

### 3.2. Shielding Test

**Figure 1:**
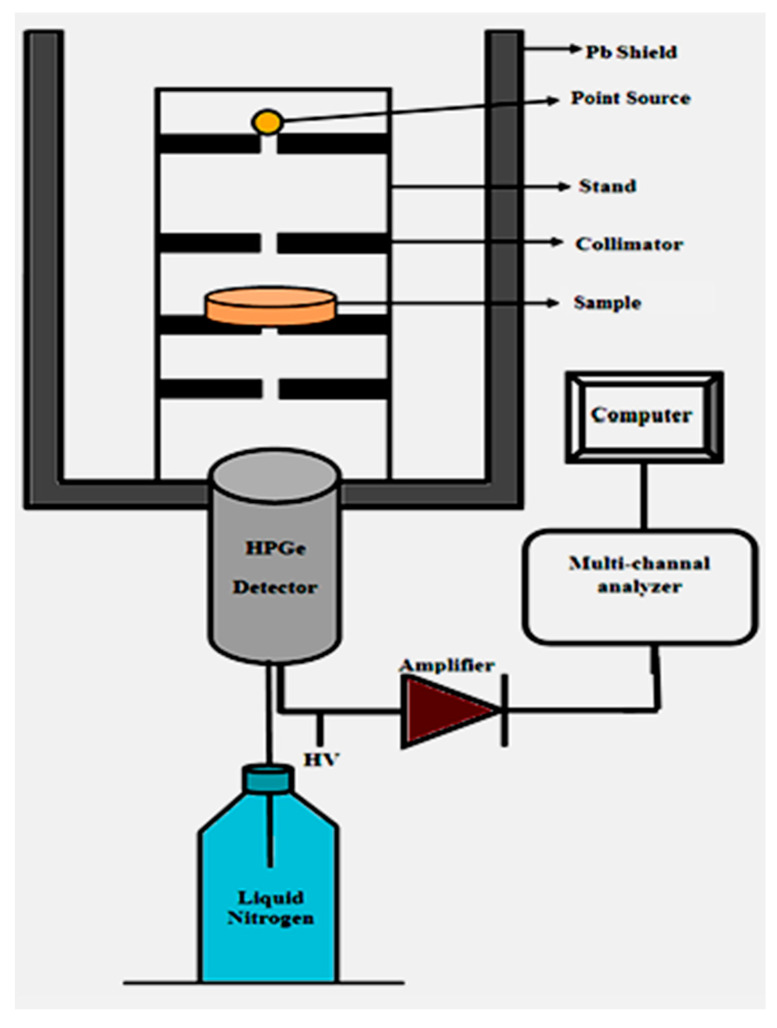
The illustration setup of the experimental work.

To achieve our goal in this work, a High Pure Germanium (HPGe) detector was used to measure the shielding factors of the prepared samples [20,21]. The detector was calibrated, and the initial count rate (N_0_) was measured in the absence of concrete; then, the prepared concrete with a certain thickness and density was placed between the detector and the source to determine the count rate (N) (see Figure 1).

The mass attenuation coefficient (MAC) was experimentally measured using Equation (1) [22]:

(1) $$MAC=\frac{1}{t\times \rho }\;ln\frac{N_{0}}{N}$$

The experimental results were compared with the results obtained from XCOM where the MAC of the composite was estimated by the following equation [23]:

(2) $$MAC=\sum_{i}w_{i}{\left(MAC\right)}_{i}$$

The linear attenuation coefficient (LAC) can be calculated by multiplying the MAC of the sample by its density, namely [24]:

(3) LAC=MAC×ρ

The half and tenth value layers were determined using Equation (4) [1,25]:

(4) $$HVL=\frac{LN\;\left(2\right)}{LAC},\;TVL=\frac{LN\;\left(10\right)}{LAC}\;$$

The radiation absorption ratio (RAR) is given by [26].

(5) $$RAR,\%=\left[1-\frac{N}{N˳}\right]\times 100$$

## 4. Results and Discussion

### 4.1. Density Results

**Figure 2:**
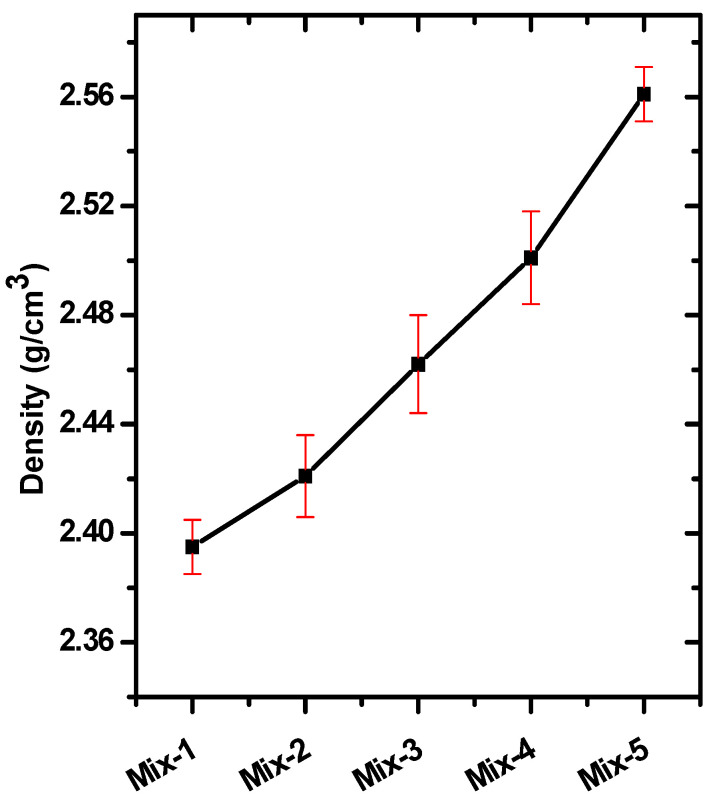
The bulk density of all prepared mixed concrete samples.

The density was measured experimentally by the law of mass over volume; the mass was measured with a sensitive balance (0.001 g) and the volume of the cylindrical sample by measuring the radius and thickness of the prepared sample. An increase in density, as shown in Figure 2, was found with the replacement of cement with red clay and the replacement of sand with crushed waste CRT glass. This is due to the high percentage of iron in red mud and high lead content in the funnel and neck of CRT glass. The densities were 2.395, 2.421, 2.462, 2.501, and 2.561 g/cm^3^ for Mix-1 to Mix-5, respectively.

### 4.2. Compressive Strength Results

**Figure 3:**
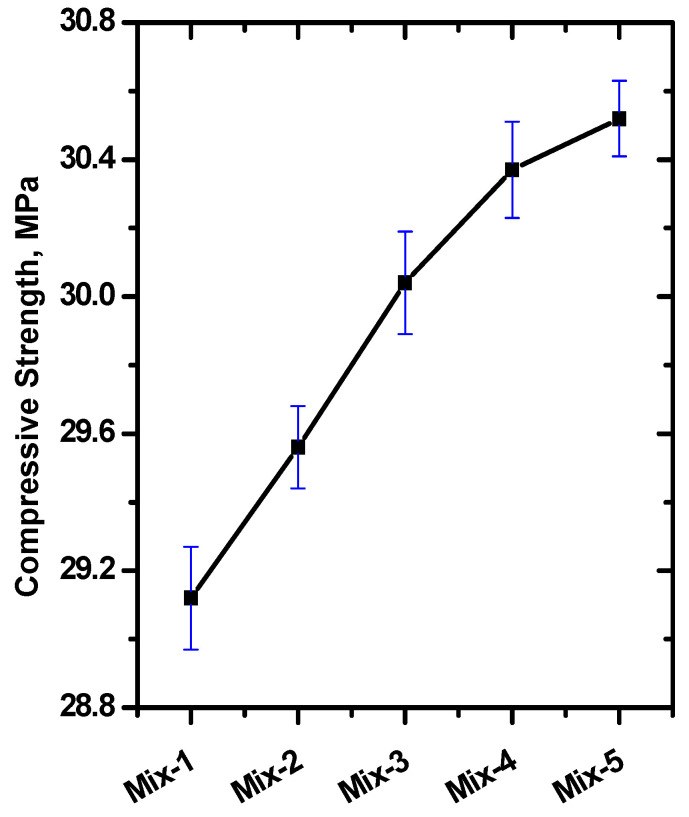
The compressive strength of all prepared mixed concrete samples.

Red mud is known for its high compressive strength. Thus, if we partially replace the cement with red clay, we increase the compressive strength of the concrete. In addition, when CRT powder is added as a partial replacement for sand, it will fill the voids in the mixture, which reduces the porosity and thus, increases the compressive strength of the mixture. Figure 3 shows the compressive strength of all prepared mixtures and the increase in compressive strength with an increase in red clay and CRT powder compared to the control mixture (Mix-1).

### 4.3. Gamma Ray Attenuation Results

**Figure 4:**
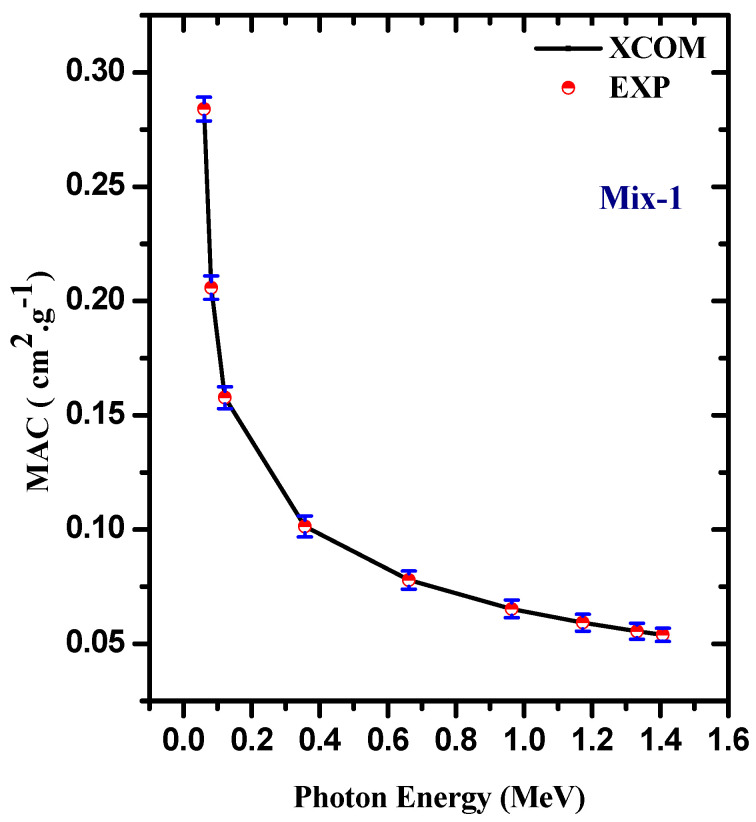
The MAC as a function of energy for both experimental and XCOM results for the ordinary concrete (Mix-1) sample.

**Figure 5:**
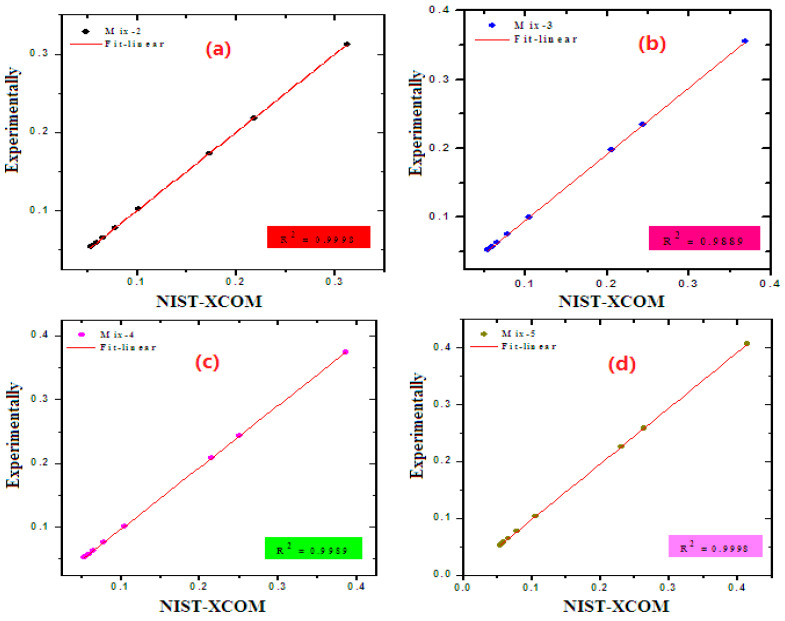
Experimental-XCOM relation of MAC for (**a**) Mix-2, (**b**) Mix-3, (**c**) Mix-4, and (**d**) Mix-5.

The mass attenuation coefficient variations of ordinary concrete (Mix-1) with different gamma energies, as measured in the laboratory and calculated by XCOM (i.e., the Theoretical approach), are depicted in Figure 4. We compared the MAC obtained by the two methods to see the degree of closeness of the measured and theoretical values. The precision of the measured MAC can be determined by calculating the deviation (Δ% = Exp-XCOM/XCOM × 100%). At any of the given energies, the experimental MAC values closely match the theoretical values and the Δ% is close to zero, confirming that the setup used in this work can effectively calculate the mass attenuation coefficients for the other prepared samples. Hence, we measured the MAC for Mix-2 through Mix-5 using the same setup and same conditions and then compared the obtained values with the XCOM as shown in Figure 5. We aimed from this step to reaffirm the degree of closeness of the measured and theoretical values, and thus, to ensure the accuracy of the results, we can use them in further calculations of other quantities through which the radiation protection properties of the samples are evaluated in this work. As we observed in the Mix-1 sample, the MAC for Mix-2 through Mix-5 obtained by both methods are good matches, which confirms the accuracy of the experimental MAC values.

**Figure 6:**
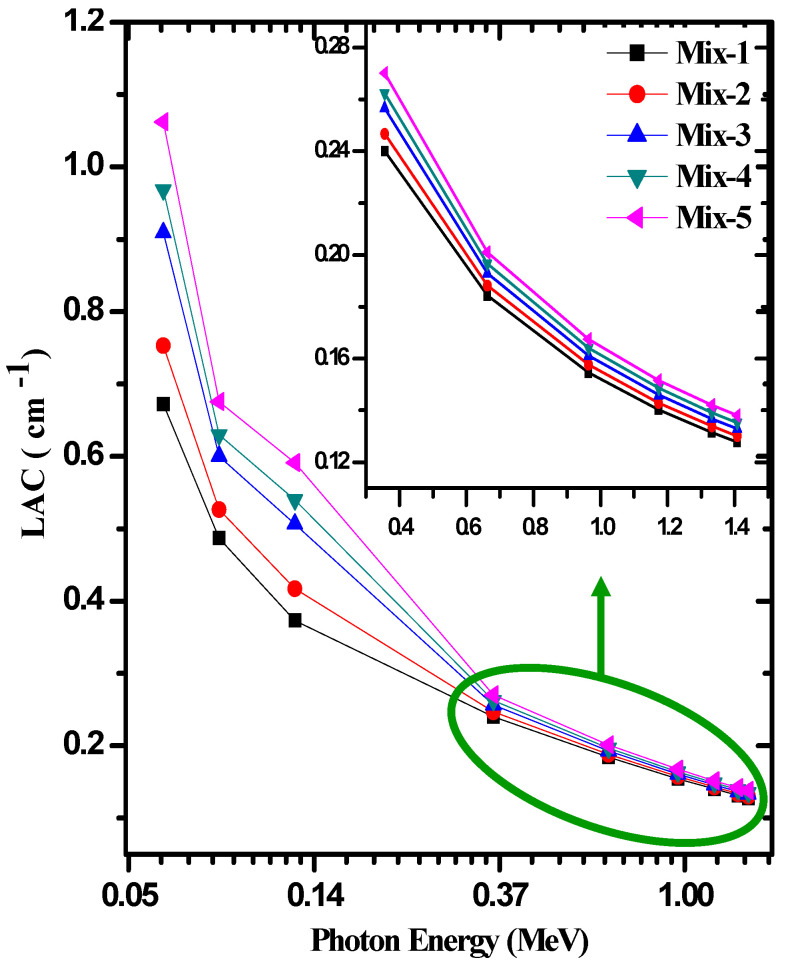
The LAC for prepared concrete samples at different energies.

In Figure 6, the linear attenuation coefficients (LAC) of the ordinary concrete (Mix-1) through Mix-5 were compared at nine different energies ranging from 0.06 to 1.408 MeV. With the increase in energy, the LAC decreased. The maximum LAC value belonged to Mix-5 at 0.06 MeV; this value was 1.062 cm^−1^. The minimum LAC value belonged to Mix-1 at 1.408 MeV; this value was 0.128 cm^−1^. These results are in line with the previous knowledge and confirm the fact that the LAC is mainly dependent on three important factors: the density of the shield, its composition, and the energy of the radiation. The radiation shielding materials developers are usually aiming to develop new materials with high LAC in order to obtain a suitable medium for radiation shielding. At 0.081 MeV, Mix-1 through Mix-5 have LAC values of 0.487, 0.526, 0.600, 0.630, and 0.676 cm^−1^. Mix-1 with zero contents of red mud and CRT glass had the lowest LAC. When we added small amounts of red mud and CRT glass (i.e., Mix-2), we found that the LAC was enhanced and the LAC for this concrete was higher than that of Mix-1. So, we concluded that the addition of red mud and CRT glass to the current composites causes an improvement in the LAC. Following this observation, we can see that as the red mud content decreases as we move from Mix-2 to Mix-5, and the CRT content increases, the LAC is enhanced. This is because the CRTs contain some heavy metals such as lead.

**Figure 7:**
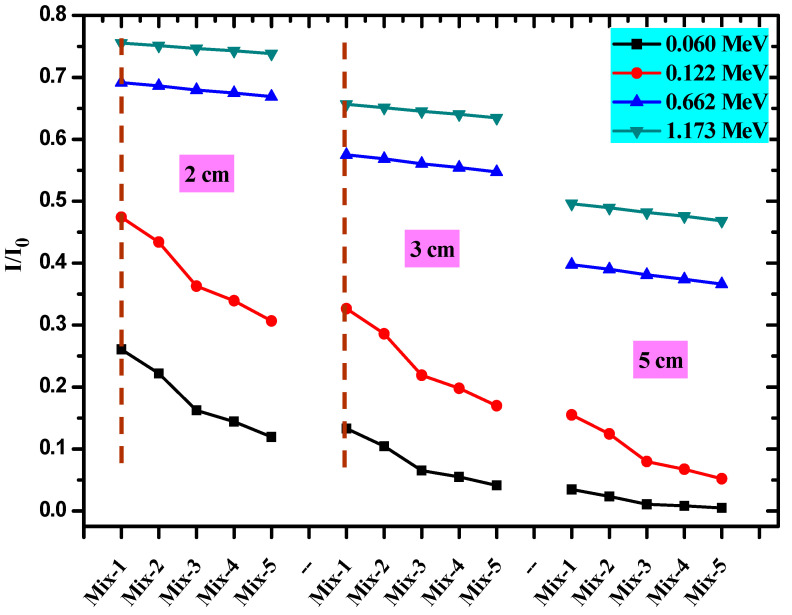
The transmission factor for all prepared concrete samples at the four energies of 0.06, 0.122, 0.662, and 1.173 MeV for the three different thicknesses 2, 3, and 5 cm.

In Figure 7, we plotted the Transmission factor for all prepared concrete at four energies, 0.06, 0.122, 0.662, and 1.173 MeV, for three different thicknesses, 2, 3, and 5 cm. We used this figure to examine the influence of the thickness of the prepared composites as well as the composition of the composites on the radiation shielding performance of the new shielding materials; we also used this figure to examine the impact of the energy of the radiation on the attenuation performance of these composites.

For a composite with any selected thickness, we found that the TF is small at 0.06 MeV, and lies in the range of 11.9–26.1% for a composite thickness of 2 cm and in the range of 4–13% for a composite thickness of 3 cm. So, the composites with a thickness of 2, 3, or 5 cm are good shields against lower energy radiation. If the energy increases, the TF is increased, and thus, the composites cannot shield most of the photons with high energies. For example, for composites with a thickness of 3 cm, we found the TF values were 57.5, 56.9, 56.1, 55.4, and 54.7% for Mix-1 through Mix-5, respectively. Thus, when a composite with a thickness of 3 cm is exposed to radiation with an energy of 0.662 MeV, about half of the photons can penetrate the composite. While, if we consider radiation with higher energy (i.e., 1.173 MeV), the TF for a layer of 3 cm thick Mix-1 through Mix-5 are 65.7, 65.1, 64.5, 64, and 63.5%. If we consider a constant thickness at a certain energy, we noticed that the TF for Mix-1 is higher than Mix-2 through Mix-5 and the lowest TF was found for Mix-5. This result demonstrated that the addition of red mud and CRTs to the current composites causes an improvement in the radiation shielding performance for the current composites which is in line with the LAC results.

**Figure 8:**
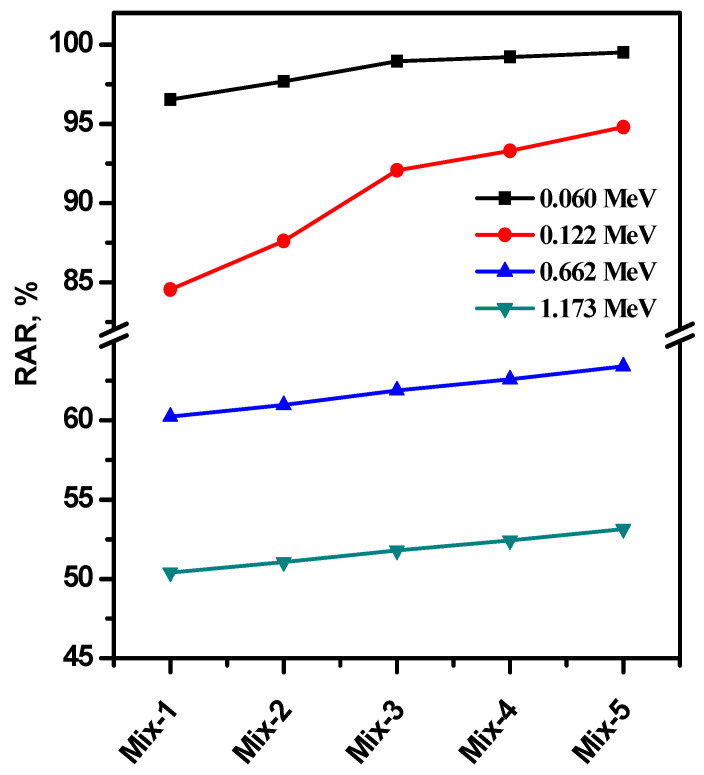
The radiation absorption ratio for all prepared concrete samples at the four energies of 0.06, 0.122, 0.662, and 1.173 MeV.

Figure 8 presents the radiation absorption ratio (RAR) for all prepared concrete samples at the four energies of 0.06, 0.122, 0.662 and 1.173 MeV. The RAR was decreased by increasing the energy. The RAR for the composites is in the range of 95–99% at 0.06 MeV, which suggests that these new composites can absorb most of the photons with an energy of 0.06 MeV; thus, these composites can be used in radiation shielding applications at low energy. At 0.122 MeV, the RAR for the composites is in the range of 85–95%, so these composites have an interesting radiation shielding ability of this low energy. As the energy increases to 0.662 MeV, we noticed that the RAR drops to 60%, while the RAR values drop to around 50–52% at 1.173 MeV. So, the new composites can shield about 50% of the radiations with high energy (i.e., 1.173 MeV). If we examine the RAR at any energy, we noticed an increase in the RAR as we move from Mix-1 to Mix-5. Thus, the increase in the CRTs in the new composites enhanced the radiation shielding features. We can thus conclude that when the CRT is maximized, more attenuation is achieved.

**Figure 9:**
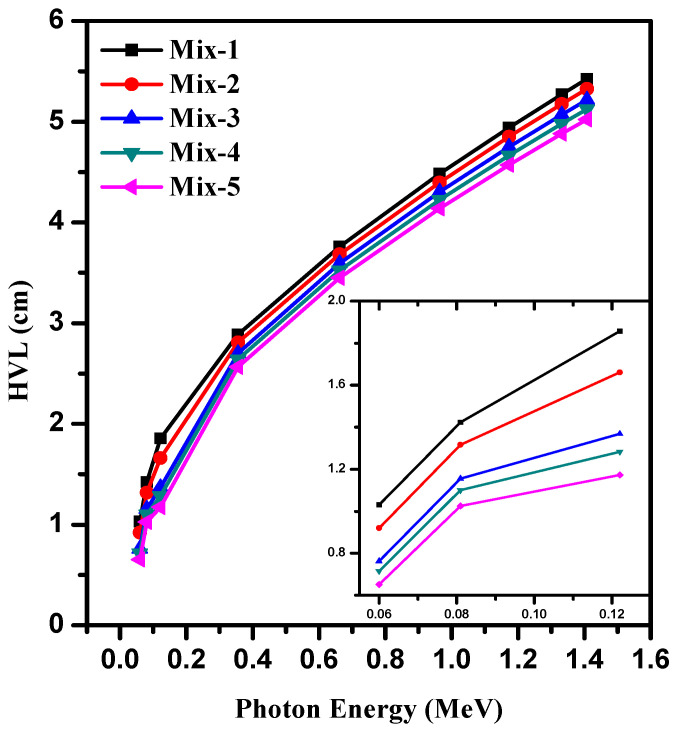
The HVL for prepared concrete samples at different energies.

In Figure 9, we presented the results for the half value layer (HVL) for the new composites. The HVL has a significant change in the energy and starts with a small value at 0.06 MeV (0.65–1.03 cm at 0.06 MeV) and reached a maximum value of about 5–5.4 cm at 1.408 MeV. If the composites are exposed to low-energy photons, a thin layer is needed to block the photons, while in the case of high-energy radiation, a thick layer is needed from the same composites. At 0.122 MeV, the HVL takes different values of 1.85, 1.66, 1.37, 1.28, and 1.17 cm for Mix-1 through Mix-5, respectively. These values become 4.48, 4.39, 4.30, 4.22, and 4.14 cm at 0.964 MeV. On the other hand, we noticed that Mix-5 has a thinner HVL than the other composites, while Mix-1 has a thicker HVL. This was correct at all energies, but we found that at higher energies, the chemical composition of the new composites did not show a significant impact on the HVL.

**Figure 10:**
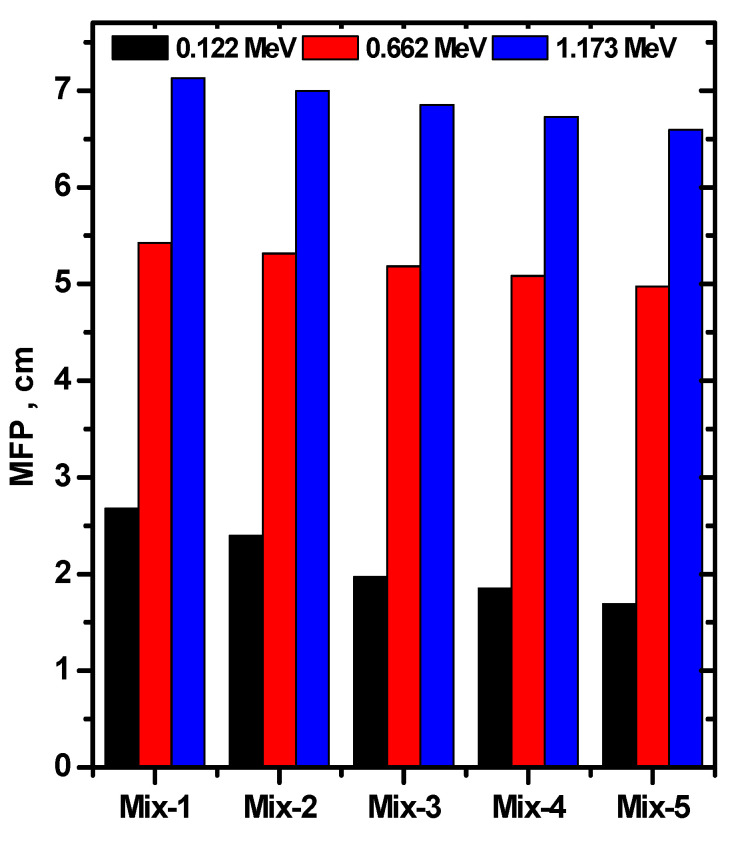
The MFP for all prepared concrete samples at 0.122, 0.662, and 1.173 MeV.

Many of the investigations on the shielding applications of composite materials have studied the impact of density on the shielding of gamma photons [27]. They found that a high-density can successfully improve the ability of a medium to absorb more photons. One of the important parameters that emphasize this fact is the mean free path (MFP). So, we evaluated the MFP for the new composites and show the results in Figure 10.

According to Figure 10, one can observe that the MFP at any energy decreases as we move from Mix-1 to Mix-5. Hence, the MFP decreases with increasing the CRT content in the new composites, as a result of increasing the density (or increasing the LAC of the composites). The lowest value of the MFP was found for Mix-5 (which contains higher amounts of CRTs). These values are 1.69, 4.97, and 6.59 cm at 0.122, 0.662, and 1.137 MeV. Accordingly, the composite with the higher CRT addition (higher density sample) may offer more interaction probability for photons and, thus, superior protection features. The difference in the MFP between the two composites Mix-1 and Mix-5 at 0.122 MeV is higher than the difference at 1.173 MeV; it is equal to 0.98 in the first case (i.e., 0.122 MeV) and 0.53 in the second case (i.e., 1.173 MeV). Therefore, we concluded that the density of the new composites plays important role in attenuating performance at low energies. The use of composites with a high CRT content is a promising method in the development of a novel radiation protective medium. According to the results of the previous figures, the utilization of a high addition of CRT into the new composites is more appropriate for medical applications with low radiation energy.

**Figure 11:**
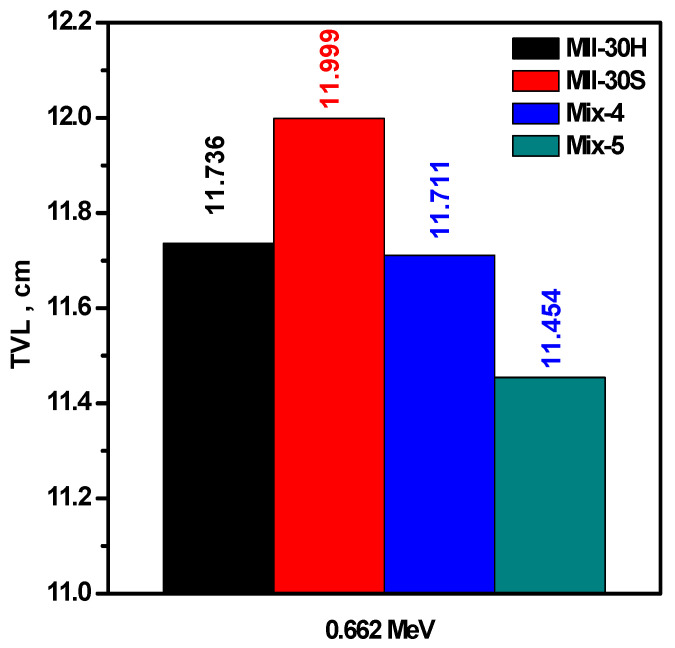
The TVL for Mix-4 and Mix-5 samples compared with concrete enhanced by coarse aggregate replacements with hematite (MII-30H) and Iron Slag (MII-30S).

To validate our findings and to show the importance of these new composites in real applications, we compared the tenth value layer (TVL) for Mix-4 and Mix-5 with hematite (MII-30H) and Iron Slag (MII-30S) partially replacing coarse aggregate [28] (see Figure 11). Mix-5 had a lower TVL than MII-30H and MII-30S, which means that Mix-5 is better than these two samples in shielding applications, while Mix-4 had almost the same TVL as MII-30H and lower TVL than MII-30S.

## 5. Conclusions

In this research, red mud and cathode ray tubes were added individually to prepare new radiation shielding concretes. The combination of red mud and CRTs has a notable impact on the radiation shielding performance of the prepared concretes. We found that the TF for Mix-1 was higher than for Mix-2 through Mix-5 and the lowest TF was found for Mix-5. This result demonstrated that the addition of red mud and CRTs to the current composites causes an improvement in the radiation shielding performance for the current composites, which is in line with the LAC results. From the HVL results, when the composites were exposed to low-energy photons, a thin layer was needed to block the photons. Mix-5 has a thinner HVL than the other composites, while Mix-1 has a thicker HVL. In addition, at higher energies, the chemical composition of the new composites did not show a significant impact on the HVL. The MFP decreases with increasing CRT content in the new composites, and the lowest value of the MFP was found for Mix-5 (which contains a higher amount of CRTs). The use of composite with high CRT content is a promising method in the development of a novel radiation protective medium. In conclusion, the utilization of a high addition of CRTs into new concrete composites is more appropriate for medical applications with low radiation energy.

## Figures and Tables

**Table 1:** The oxide composition of cement and red mud.

Oxide Composition (%)										
	Al_2_O_3_	SiO_2_	Fe_2_O_3_	CaO	MgO	TiO_2_	SO_3_	K_2_O	Na_2_O	L.O.I
Cement	4.88	21.38	5.86	58.22	2.66	—	2.87	0.32	0.41	3.40
Red mud	22.31	19.22	34.89	14.11	0.33	4.55	—	—	3.11	1.51

**Table 2:** Physical properties of the aggregates used in this investigation.

	Fine Aggregate	Course Aggregate	CRT Glass
Relative density	2.59	2.63	3.15
Loose Unit Weight (t/m^3^)	1.54	1.33	1.22
Hardness index	6.22	6.89	—

**Table 3:** Chemical compositions of the aggregates used in this investigation.

Oxides	Chemical Composition (wt%)		
	Sand	Limestone	CRT Glass
SiO_2_	99.38	14.53	51.24
Al_2_O_3_	—	0.72	4.02
Fe_2_O_3_	—	0.41	—
CaO	0.01	42.85	3.81
MgO	—	2.18	1.82
SO_3_	0.04	0.03	—
K_2_O	0.07	—	7.81
Na_2_O	—	—	6.76
BaSO_4_	—	—	—
SrO	—	—	0.51
PbO	—	—	22.18
BaO	—	—	0.52
Sb_2_O_3_	—	—	0.28
L.O.I	0.5	39.28	1.05

**Table 4:** Concrete mix proportions.

Mix Name	Cement (Kg/m^3^)	Red Mud (Kg/m^3^)	Water (Kg/m^3^)	Sand (Kg/m^3^)	CRT Glass (Kg/m^3^)	Limestone (Kg/m^3^)	SP (Kg/m^3^)
Mix-l	400	—	180	830.0	—	995.0	9.8
Mix-2	360	40.2	180	747.9	83.4	996.2	8.2
Mix-3	320	81.5	180	671.7	162.2	998.5	7.9
Mix-4	280	122.1	180	593.5	243.5	999.2	8.8
Mix-5	240	163.2	180	502.3	332.4	999.8	9.6

## Data Availability

Not applicable.
